# Europium-doped barium bromide iodide

**DOI:** 10.1107/S1600536809041105

**Published:** 2009-10-17

**Authors:** Gautam Gundiah, Stephen M. Hanrahan, Frederick J. Hollander, Edith D. Bourret-Courchesne

**Affiliations:** a Life Sciences Division, Lawrence Berkeley National Laboratory, Berkeley, CA 94720, USA; b Department of Chemistry, University of California, Berkeley, CA 94720, USA; c Materials Sciences Division, Lawrence Berkeley National Laboratory, Berkeley, CA 94720, USA

## Abstract

Single crystals of Ba_0.96_Eu_0.04_BrI (barium europium bromide iodide) were grown by the Bridgman technique. The title compound adopts the ordered PbCl_2_ structure [Braekken (1932 ▶). *Z. Kristallogr.* **83**, 222–282]. All atoms occupy the fourfold special positions (4*c*, site symmetry *m*) of the space group *Pnma* with a statistical distribution of Ba and Eu. They lie on the mirror planes, perpendicular to the *b* axis at *y* = ±0.25. Each cation is coordinated by nine anions in a tricapped trigonal prismatic arrangement.

## Related literature

For details of crystal growth by the Bridgman technique, see: Robertson (1986 ▶). For structural details of isotypic compounds, see: PbCl_2_ (Braekken, 1932 ▶); EuBrI (Liao *et al.*, 2004 ▶); SrBrI (Hodorowicz & Eick, 1983 ▶); and BaBrCl (Hodorowicz *et al.*, 1983 ▶). For structural details of PbFCl compounds, see: Liebich & Nicollin (1977 ▶). For the structure of compounds with similar compositions by powder diffraction, see Lenus *et al.* (2002 ▶). For the luminescent properties of some Eu^2+^-activated barium halides, see: Schweizer (2001 ▶); Crawford & Brixner (1991 ▶); Selling *et al.* (2007 ▶); Bourret-Courchesne *et al.* (2009 ▶).

## Experimental

### 

#### Crystal data

Ba_0.96_Eu_0.04_BrI
*M* *_r_* = 344.70
Orthorhombic, 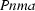

*a* = 8.684 (3) Å
*b* = 5.0599 (19) Å
*c* = 10.061 (4) Å
*V* = 442.1 (3) Å^3^
*Z* = 4
Mo *K*α radiation
μ = 24.97 mm^−1^
*T* = 153 K
0.14 × 0.09 × 0.06 mm

#### Data collection

Bruker SMART 1000 CCD diffractometer
Absorption correction: multi-scan (Blessing, 1995 ▶) *T*_min_ = 0.128, *T*_max_ = 0.316
2609 measured reflections
430 independent reflections
370 reflections with *I* > 2σ(*I*)
*R*_int_ = 0.027

#### Refinement

*R*[*F* ^2^ > 2σ(*F* ^2^)] = 0.015
*wR*(*F* ^2^) = 0.033
*S* = 1.02
430 reflections
21 parameters
Δρ_max_ = 0.89 e Å^−3^
Δρ_min_ = −0.78 e Å^−3^

### 

Data collection: *APEX2* (Bruker, 2008 ▶); cell refinement: *SAINT* (Bruker, 2008 ▶); data reduction: *SAINT*; program(s) used to solve structure: *SHELXS97* (Sheldrick, 2008 ▶); program(s) used to refine structure: *SHELXL97* (Sheldrick, 2008 ▶); molecular graphics: *SHELXTL* (Sheldrick, 2008 ▶); software used to prepare material for publication: *SHELXTL*.

## Supplementary Material

Crystal structure: contains datablocks I, global. DOI: 10.1107/S1600536809041105/fi2082sup1.cif

Structure factors: contains datablocks I. DOI: 10.1107/S1600536809041105/fi2082Isup2.hkl

Additional supplementary materials:  crystallographic information; 3D view; checkCIF report

## Figures and Tables

**Table 1:** Selected bond lengths (Å)

I1—Ba1^i^	3.6448 (10)
I1—Ba1^ii^	3.6448 (10)
I1—Ba1^iii^	3.7101 (9)
I1—Ba1^iv^	3.7101 (9)
Ba1—Br1^v^	3.2643 (10)
Ba1—Br1	3.2643 (10)
Ba1—Br1^vi^	3.2931 (13)
Ba1—Br1^vii^	3.3065 (14)

Symmetry codes: (i) 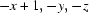; (ii) 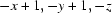; (iii) 
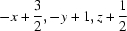
; (iv) 
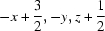
; (v) 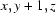; (vi) 
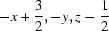
; (vii) 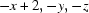.

**Fig. 1.**
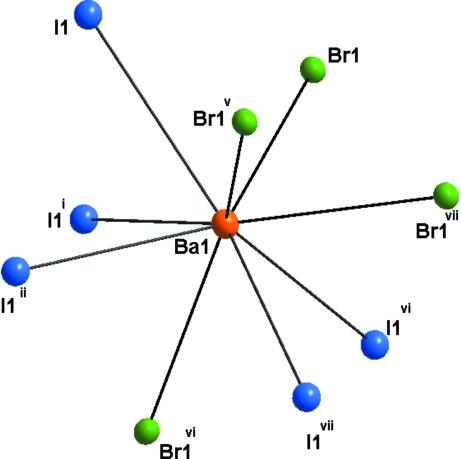
Arrangement of anions around each Ba atom. The displacement ellipsoids are given at 50% probability. The symmetry codes are: (i) -x + 1, -y, -z; (ii) -x + 1, -y + 1, -z; (iii) -x + 3/2, -y + 1, z + 1/2; (iv) -x + 3/2, -y, z + 1/2; (v) x, y + 1, z; (vi) -x + 3/2, -y, z - 1/2; (vii) -x + 2, -y, -z; (viii) -x + 3/2, -y + 1, z - 1/2; (ix) x, y - 1, z.

## supplementary crystallographic information

### Comment

Barium mixed halides activated by Eu^2+^ have been extensively studied as X-ray phosphors (Schweizer, 2001; Crawford & Brixner, 1991) and scintillators for the detection of γ-rays (Selling *et al.*, 2007). The F-based compounds of the form BaFX (*X*= Cl, Br, I) have a tetragonal, matlockite structure similar to PbFCl (Liebich & Nicollin, 1977). Among the other barium mixed halides, the structure of BaBrCl has been found to be the PbCl_2_-type (Hodorowicz *et al.*, 1983). Lenus *et al.* recently solved the structures of BaBrI and BaClI from X-ray powder diffraction data in the space groups *P222_1_* and *Pbam* respectively (Lenus *et al.*, 2002). We have synthesized single crystals of Ba_0.96_Eu_0.04_BrI and present details of the structure. Eu is introduced as a dopant and substitute for Ba. The doping was not expected to change the structure of the parent material BaBrI. However, we determine the structure to have a space group *Pnma, *similar to that of isomorphous compounds EuBrI (Liao *et al.*, 2004) and SrBrI (Hodorowicz & Eick, 1983), but not the structure published by Lenus *et al.* for powders of BaBrI (Lenus *et al.*, 2002)

The title compound adopts the orthorhombic PbCl_2_ structure. All atoms occupy the fourfold special positions (4c) of the space group *D^16^_2h_-Pnma*. They lie on the mirror planes, perpendicular to the *b* axis at *y* = (±)0.25. Each Ba/Eu cation is coordinated by 9 anions in a tricapped trigonal prismatic arrangement (Fig. 1). The anions are not equidistant from the Ba cation but present in two different positions. The smaller bromide anions occupy one of the anionic positions at distances between 3.26 and 3.30 Å. The larger iodide anions occupy the second anionic position (distances 3.62 - 3.71 Å), giving a completely ordered structure for the anions. The same ordering has been observed in isomorphous compounds EuBrI (Liao *et al.*, 2004) and SrBrI (Hodorowicz & Eick, 1983).

The Eu content of 4% has been determined from the refinement of the structure. The presence of divalent Eu is also confirmed by measuring the emission curve under X-ray excitation. The characteristic *4f^6^5 d^1 ^*→*4f^7^*transition of Eu^2+^ was observed. A detailed study of the luminescent properties is currently underway and will be presented in a future publication (Bourret-Courchesne *et al.*, 2009).

### Experimental

Single crystals with the composition Ba_0.96_Eu_0.04_BrI were grown by the vertical Bridgman techniques. BaBr_2_, BaI_2_, EuBr_2_ and EuI_2_ were obtained commerically, mixed in the molar ratio 0.48: 0.48: 0.02: 0.02 and sealed in a quartz ampoule under a dynamic vacuum of 1.10 ^–6^ Torr. The sealed ampoule, about 1 cm in diameter, was heated in a 24 zone Mellen furnace to a temperature of 1123 K and directionally cooled to provide a growth rate of 1 mm/hour. The reactants and products are moisture-sensitive and all manipulations were carried out inside an Argon-filled glove box. The crystal obtained is colorless.

### Refinement

The doping of Eu(ii) on the Ba(ii) site was modeled with a fractional Eu atom fixed in the same location and with the same thermal parameters as the Ba(ii) atom. The relative occupancy factor refined to 0.963 (13) Ba, 0.037 (13) Eu.

### Crystal data

**Table:**

Ba_0.96_Eu_0.04_BrI	*F*(000) = 576.7
*M**_r_* = 344.70	*D*_x_ = 5.179 Mg m^−^^3^
Orthorhombic, *P**n**m**a*	Mo *K*α radiation, λ = 0.71073 Å
Hall symbol: -P 2ac 2n	Cell parameters from 1548 reflections
*a* = 8.684 (3) Å	θ = 4.5–25.4°
*b* = 5.0599 (19) Å	µ = 24.97 mm^−^^1^
*c* = 10.061 (4) Å	*T* = 153 K
*V* = 442.1 (3) Å^3^	Block, colourless
*Z* = 4	0.14 × 0.09 × 0.06 mm

### Data collection

**Table:**

Bruker SMART 1000 CCD diffractometer	430 independent reflections
Radiation source: fine-focus sealed tube	370 reflections with *I* > 2σ(*I*)
graphite	*R*_int_ = 0.027
Detector resolution: 8.192 pixels mm^-1^	θ_max_ = 25.0°, θ_min_ = 3.1°
φ and ω scans	*h* = −9→10
Absorption correction: multi-scan (Blessing, 1995)	*k* = −6→5
*T*_min_ = 0.128, *T*_max_ = 0.316	*l* = −11→11
2609 measured reflections	

### Refinement

**Table:**

Refinement on *F*^2^	Primary atom site location: heavy-atom method
Least-squares matrix: full	*w* = 1/[σ^2^(*F*_o_^2^) + (0.02*P*)^2^] where *P* = (*F*_o_^2^ + 2*F*_c_^2^)/3
*R*[*F*^2^ > 2σ(*F*^2^)] = 0.015	(Δ/σ)_max_ = 0.001
*wR*(*F*^2^) = 0.033	Δρ_max_ = 0.89 e Å^−^^3^
*S* = 1.01	Δρ_min_ = −0.78 e Å^−^^3^
430 reflections	Extinction correction: *SHELXL97* (Sheldrick, 2008), Fc^*^=kFc[1+0.001xFc^2^λ^3^/sin(2θ)]^-1/4^
21 parameters	Extinction coefficient: 0.0151 (5)
0 restraints	

### Special details

**Table:**

Geometry. All e.s.d.'s (except the e.s.d. in the dihedral angle between two l.s. planes) are estimated using the full covariance matrix. The cell e.s.d.'s are taken into account individually in the estimation of e.s.d.'s in distances, angles and torsion angles; correlations between e.s.d.'s in cell parameters are only used when they are defined by crystal symmetry. An approximate (isotropic) treatment of cell e.s.d.'s is used for estimating e.s.d.'s involving l.s. planes.
Refinement. Refinement of *F*^2^ against ALL reflections. The weighted *R*-factor *wR* and goodness of fit *S* are based on *F*^2^, conventional *R*-factors *R* are based on *F*, with *F* set to zero for negative *F*^2^. The threshold expression of *F*^2^ > σ(*F*^2^) is used only for calculating *R*-factors(gt) *etc*. and is not relevant to the choice of reflections for refinement. *R*-factors based on *F*^2^ are statistically about twice as large as those based on *F*, and *R*- factors based on ALL data will be even larger.The doping of Eu(ii) on the Ba(ii) site was modeled with a fractional Eu atom fixed in the same location and with the same thermal parameters as the Ba(ii) atom. The relative occupancy factor refined to 0.963 (13) Ba, 0.037 (13) Eu.

### Fractional atomic coordinates and isotropic or equivalent isotropic displacement parameters (Å^2^)

**Table:**

	*x*	*y*	*z*	*U*_iso_*/*U*_eq_	Occ. (<1)
I1	0.52804 (5)	0.2500	0.16976 (4)	0.01285 (18)	
Ba1	0.76955 (4)	0.2500	−0.12472 (4)	0.01213 (17)	0.963 (13)
Eu1	0.76955 (4)	0.2500	−0.12472 (4)	0.01213 (17)	0.037 (13)
Br1	0.85573 (8)	−0.2500	0.06634 (6)	0.0107 (2)	

### Atomic displacement parameters (Å^2^)

**Table:**

	*U*^11^	*U*^22^	*U*^33^	*U*^12^	*U*^13^	*U*^23^
I1	0.0119 (3)	0.0139 (3)	0.0128 (2)	0.000	0.00011 (16)	0.000
Ba1	0.0110 (3)	0.0126 (3)	0.0128 (2)	0.000	−0.00069 (15)	0.000
Eu1	0.0110 (3)	0.0126 (3)	0.0128 (2)	0.000	−0.00069 (15)	0.000
Br1	0.0096 (4)	0.0119 (5)	0.0105 (3)	0.000	−0.0008 (2)	0.000

### Geometric parameters (Å, °)

**Table:**

I1—Ba1	3.6299 (11)	Ba1—Br1^vii^	3.3065 (14)
I1—Ba1^i^	3.6448 (10)	Ba1—I1^i^	3.6448 (10)
I1—Ba1^ii^	3.6448 (10)	Ba1—I1^ii^	3.6448 (10)
I1—Ba1^iii^	3.7101 (9)	Ba1—I1^vi^	3.7101 (9)
I1—Ba1^iv^	3.7101 (9)	Ba1—I1^viii^	3.7101 (9)
Ba1—Br1^v^	3.2643 (10)	Br1—Ba1^ix^	3.2643 (10)
Ba1—Br1	3.2643 (10)	Br1—Ba1^iv^	3.2930 (13)
Ba1—Br1^vi^	3.2931 (13)	Br1—Ba1^vii^	3.3065 (14)
			
Ba1—I1—Ba1^i^	107.950 (19)	Br1—Ba1—I1^ii^	140.69 (2)
Ba1—I1—Ba1^ii^	107.950 (19)	Br1^vi^—Ba1—I1^ii^	69.416 (14)
Ba1^i^—I1—Ba1^ii^	87.92 (3)	Br1^vii^—Ba1—I1^ii^	136.041 (16)
Ba1—I1—Ba1^iii^	100.44 (2)	I1—Ba1—I1^ii^	72.051 (19)
Ba1^i^—I1—Ba1^iii^	151.463 (16)	I1^i^—Ba1—I1^ii^	87.92 (3)
Ba1^ii^—I1—Ba1^iii^	86.09 (3)	Br1^v^—Ba1—I1^vi^	138.42 (2)
Ba1—I1—Ba1^iv^	100.44 (2)	Br1—Ba1—I1^vi^	72.00 (3)
Ba1^i^—I1—Ba1^iv^	86.09 (3)	Br1^vi^—Ba1—I1^vi^	68.31 (2)
Ba1^ii^—I1—Ba1^iv^	151.463 (16)	Br1^vii^—Ba1—I1^vi^	68.457 (18)
Ba1^iii^—I1—Ba1^iv^	85.99 (3)	I1—Ba1—I1^vi^	136.767 (14)
Br1^v^—Ba1—Br1	101.62 (3)	I1^i^—Ba1—I1^vi^	78.07 (2)
Br1^v^—Ba1—Br1^vi^	129.163 (17)	I1^ii^—Ba1—I1^vi^	137.726 (19)
Br1—Ba1—Br1^vi^	129.163 (17)	Br1^v^—Ba1—I1^viii^	72.00 (3)
Br1^v^—Ba1—Br1^vii^	70.719 (16)	Br1—Ba1—I1^viii^	138.42 (2)
Br1—Ba1—Br1^vii^	70.718 (16)	Br1^vi^—Ba1—I1^viii^	68.31 (2)
Br1^vi^—Ba1—Br1^vii^	119.523 (18)	Br1^vii^—Ba1—I1^viii^	68.457 (18)
Br1^v^—Ba1—I1	69.62 (2)	I1—Ba1—I1^viii^	136.767 (14)
Br1—Ba1—I1	69.62 (2)	I1^i^—Ba1—I1^viii^	137.726 (19)
Br1^vi^—Ba1—I1	125.42 (3)	I1^ii^—Ba1—I1^viii^	78.07 (2)
Br1^vii^—Ba1—I1	115.06 (2)	I1^vi^—Ba1—I1^viii^	85.99 (3)
Br1^v^—Ba1—I1^i^	140.69 (2)	Ba1—Br1—Ba1^ix^	101.62 (3)
Br1—Ba1—I1^i^	72.41 (2)	Ba1^ix^—Br1—Ba1^iv^	118.69 (2)
Br1^vi^—Ba1—I1^i^	69.416 (14)	Ba1—Br1—Ba1^vii^	109.282 (16)
Br1^vii^—Ba1—I1^i^	136.041 (16)	Ba1^ix^—Br1—Ba1^vii^	109.282 (16)
I1—Ba1—I1^i^	72.051 (19)	Ba1^iv^—Br1—Ba1^vii^	99.06 (2)
Br1^v^—Ba1—I1^ii^	72.41 (2)		

Symmetry codes: (i) −*x*+1, −*y*, −*z*; (ii) −*x*+1, −*y*+1, −*z*; (iii) −*x*+3/2, −*y*+1, *z*+1/2; (iv) −*x*+3/2, −*y*, *z*+1/2; (v) *x*, *y*+1, *z*; (vi) −*x*+3/2, −*y*, *z*−1/2; (vii) −*x*+2, −*y*, −*z*; (viii) −*x*+3/2, −*y*+1, *z*−1/2; (ix) *x*, *y*−1, *z*.

### Table 2 Selected geometric parameters (Å, °)

**Table:**

Ba1—I1^i^	3.6448 (10)	Ba1—I1^ii^	3.6448 (10)
Ba1—I1^vi^	3.7101 (9)	Ba1—I1^viii^	3.7101 (9)
Ba1—Br1^vii^	3.3065 (14)	Ba1—Br1^v^	3.2643 (10)
Ba1—Br1	3.2643 (10)	Ba1—Br1^vi^	3.2931 (13)
Br1^v^—Ba1—Br1	101.62 (3)	Br1—Ba1—I1^ii^	140.69 (2)
Br1^v^—Ba1—Br1^vi^	129.163 (17)	Br1^vi^—Ba1—I1^ii^	69.416 (14)
Br1—Ba1—Br1^vi^	129.163 (17)	Br1^vii^—Ba1—I1^ii^	136.041 (16)
Br1^v^—Ba1—Br1^vii^	70.719 (16)	Br1^v^—Ba1—I1^vi^	.138.42 (2)
Br1—Ba1—Br1^vii^	70.718 (16)	Br1—Ba1—I1	69.62 (2)
Br1^vi^—Ba1—Br1^vii^	119.523 (18)	Br1^v^—Ba1—I1	69.62 (2)
I1^i^—Ba1—I1^ii^	87.92 (3)	Br1—Ba1—I1^vi^	72.00 (3)
I1—Ba1—I1^vi^	136.767 (14)	Br1^vi^—Ba1—I1^vi^	68.31 (2)
I1^i^—Ba1—I1^vi^	78.07 (2)	Br1^vii^—Ba1—I1^vi^	68.457 (18)
I1^ii^—Ba1—I1^vi^	137.726 (19)	Br1^vi^—Ba1—I1	125.42 (3)
.I1—Ba1—I1^ii^	.72.051 (19)	Br1^vii^—Ba1—I1	115.06 (2)
Br1—Ba1—I1^i^	72.41 (2)	Br1^v^—Ba1—I1^i^	140.69 (2)
I1—Ba1—I1^i^	72.051 (19)	Br1^vi^—Ba1—I1^i^	69.416 (14)
Br1^vii^—Ba1—I1^i^	136.041 (16)	Br1^v^—Ba1—I1^ii^	.72.41 (2)

Symmetry codes: (i) -x+1, -y, -z; (ii) -x+1, -y+1, -z; (iii) -x+3/2, -y+1, z+1/2; (iv) -x+3/2, -y, z+1/2; (v) x, y+1, z; (vi) -x+3/2, -y, z-1/2; (vii) -x+2, -y, -z; (viii) -x+3/2, -y+1, z-1/2; (ix) x, y-1, z.

## References

[bb1] Blessing, R. H. (1995). *Acta Cryst.* A**51**, 33–38. 10.1107/s0108767394005726 7702794

[bb2] Bourret-Courchesne, E. D., Gundiah, G., Hanrahan, S. M., Bizarri, G. & Derenzo, S. E. (2009). In preparation.

[bb3] Braekken, H. (1932). *Z. Kristallogr.* **83**, 222–282.

[bb4] Bruker (2008). *APEX2* and *SAINT* Bruker AXS Inc., Madison, Wisconsin, USA.

[bb5] Crawford, M. K. & Brixner, L. H. (1991). *J. Lumin.* **48**–**49**, 37–42.

[bb6] Hodorowicz, S. A. & Eick, H. A. (1983). *J. Solid State Chem.* **46**, 313–320.

[bb7] Hodorowicz, S. A., Hodorowicz, E. K. & Eick, H. A. (1983). *J. Solid State Chem.* **48**, 351–356.

[bb8] Lenus, A. J., Sornadurai, D., Rajan, K. G. & Purniah, B. (2002). *Powder Diffr.* **17**, 331–335.

[bb9] Liao, W., Liu, X. & Dronskowski, R. (2004). *Acta Cryst.* E**60**, i69–i71. 10.1107/S0108270103028671 15004351

[bb10] Liebich, B. W. & Nicollin, D. (1977). *Acta Cryst.* B**33**, 2790–2794.

[bb11] Robertson, J. M. (1986). *Crystal Growth of Ceramics: Bridgman–Stockbarger Methods*, pp. 963–964. In *Encyclopedia of Materials Science and Engin­eering*, edited by M. B. Bever. Oxford: Pergamon.

[bb12] Schweizer, S. (2001). *Phys. Status Solidi A*, **187**, 335–393.

[bb13] Selling, J., Birowosuto, M. D., Dorenbos, P. & Schweizer, S. (2007). *J. Appl. Phys.* **101**, 034901.

[bb14] Sheldrick, G. M. (2008). *Acta Cryst.* A**64**, 112–122. 10.1107/S0108767307043930 18156677

